# Health-Related Rumor Control through Social Collaboration Models: Lessons from Cases in China during the COVID-19 Pandemic

**DOI:** 10.3390/healthcare10081475

**Published:** 2022-08-05

**Authors:** Feng Yang, Yunyue Ren, Shusheng Wang, Xiaoqian Zhang

**Affiliations:** 1 School of Public Administration, Sichuan University, Chengdu 610065, China; 2 School of Information Studies, McGill University, Montreal, QC H3A 1X1, Canada

**Keywords:** health rumors, rumor control, rumor-debunking model, content analysis, pandemic, COVID-19, China

## Abstract

Widely spread health-related rumors may mislead the public, escalate social panic, compromise government credibility, and threaten public health. Social collaboration models that maximize the functions and advantages of various agents of socialization can be a promising way to control health-related rumors. Existing research on health-related rumors, however, is limited in studying how various agents collaborate with each other to debunk rumors. This study utilizes content analysis to code the text data of health-related rumor cases in China during the COVID-19 pandemic. The study found that socialized rumor-debunking models could be divided into the following five categories: the government-led model, the media-led model, the scientific community-led model, the rumor-debunking platform-led model, and the multi-agent collaborative model. In addition, since rumors in public health crises often involve different objects, rumor refutation requires various information sources; therefore, different rumor-debunking models apply. This study verifies the value of socialized collaborative rumor debunking, advocates and encourages the participation of multiple agents of socialization and provides guidance for establishing a collaborative rumor-debunking model, thereby promoting efficient rumor-debunking methods and improving the healthcare of society.

## 1. Introduction

Rumors are regarded as one of the oldest sources of information. The study of rumors may be traced back to World War II, when American sociologist Knapp defined a rumor as a proposition for belief of topical reference disseminated without official verification [1]. Since then, rumors have been studied from the perspectives of sociology, psychology, and communication [2]. Rumors can be either dis-information or misinformation; that is, they can be false information that is spread deliberately or erroneous information that is distorted during transmittance.

Although rumors were initially spread by word of mouth [3], the advent of social media channels with large audiences, interactiveness, and synchrony has accelerated their spread. In the information age, everyone is allowed to create or transmit information; rumors, which used to be spread by word of mouth, are now spreading even more rapidly via the Internet, thereby reaching a wider audience and causing more harm to society. Social media has been widely used to inform people about the progress of crises or disasters, and rumors often coexist when disseminating general information [4]. Moreover, rumors usually diffuse more rapidly and widely than the truth [5].

The COVID-19 pandemic has significantly changed the whole world. As a country where the virus was first reported, China has received a lot of attention in academia. For example, past literature has discussed mental health issues during the pandemic [6,7], the impact of lockdown policies on different industries [8,9] and vaccination-related issues among various populations [10,11,12].

Rumor control is also an important issue to address during the COVID-19 pan-demic. A considerable number of rumors about virus protection, pandemic prevention, and new cases could be found on social media during the COVID-19 pandemic [13,14,15,16]. These widespread health-related rumors have significantly affected global health [17], so much so that debunking health-related rumors has become as important as fighting the pandemic. Failing to refute rumors and issue an authoritative response in such times may mislead the public, escalate social panic, compromise government credibility, and threaten public health.

The government used to be responsible for preventing and controlling rumors. People simply wait for governments to debunk rumors [18]. However, this traditional model is limited in dealing with health-related rumors. People’s level of trust and confidence in the government place a significant role in health-related situations [19]. The governments also need more time to respond to many sudden public health crises because of the lack of medical knowledge [18]. Social collaboration models can be a promising way to control health-related rumors. These models maximize the functions and advantages of various agents of socialization; in other words, the government, the media, network companies, social organizations, and netizens (i.e., people who regularly use the Internet) all should actively engage in controlling health-related rumors through information exchange, resource sharing, and collaborative decision-making.

Research on controlling rumors has found various advantages in involving multiple agents (such as governments, social organizations, and media companies) and suggests that different agents should utilize their distinctive advantages and adopt different strategies, depending on the types of rumors [17,20]. For example, governments are suggested to provide clear information via official accounts, design programs to improve the public’s knowledge, and take legal action against spreading rumors [21,22,23]. Healthcare organizations, such as the World Health Organization (WHO), are expected to provide professional information for rumor refutation [17]. Social media companies should also take the responsibility of reviewing health-related information on their platforms [24,25]. Health professionals are credible and authoritative information sources, and therefore play vital roles in conveying the right information to their patients [26,27].

However, to the authors’ knowledge, existing research on health-related rumors is limited in studying how various agents collaborate with each other. In other words, although they may have noticed the benefits of involving the media, experts and scholars, the public, and other agents in controlling health-related rumors [28,29,30], they have missed the methods of collaboration among these agents. Past research is more interested in exploring the strength of each agent in debunking rumors rather than the collaboration between them [31]. For example, compared to the media, health professionals have been found more to be effective in communicating health information during a public health emergency [26].

During the process of debunking rumors through social collaboration, whether and how to collaborate is of particular importance. Therefore, this study aimed to (1) identify the composition of rumor-debunking agents and their collaboration, (2) summarize the socialized collaborative rumor-debunking models employed during the COVID-19 pandemic, and (3) propose a series of measures to support the operation of social collaboration models.

## 2. Materials and Methods

### 2.1. Data Source

A search for health-related rumors was conducted on the Chinese collaborative rumor-debunking online platform (www.piyao.org.cn) (accessed on 14 March 2021) in March 2021. This platform was jointly launched by the Cyberspace Administration of China, the media, and online platforms. This platform collects all types of rumors from across the nation that have been authoritatively refuted since the outbreak of the COVID-19 pandemic.

We did not limit the specific types of rumors. However, these rumors must have appeared between 20 January and 30 June 2020, during which the COVID-19 pandemic in China went through the stages of an initial outbreak, the full spread, effective containment, and regular pandemic prevention and control. It is also the period with the largest number of rumors. A total of 514 rumors were collected at this time.

To ensure the accuracy of the data, we adopted the following exclusion criteria: (1) removing duplicated cases, (2) removing rumors that had not been debunked, and (3) removing vague cases. In the end, 354 cases of health-related rumors were obtained for analysis. For each included rumor, the following parameters were recorded: (1) information source, (2) the release time, (3) the original version of the rumor, and (4) the specific rumor-debunking process.

### 2.2. Methods

Content analysis was conducted in this study. Content analysis is designed in order to elucidate “what they mean to people, what they enable or prevent, and what the information conveyed by them does” [32] (p. 2). This paper aims to uncover rumor-debunking agents and their collaboration. So, we first converted the collected rumor cases into themes by analyzing their content/texts. Then, we analyzed the relationship of these themes to identify the social collaboration models in the debunking health-related rumors.

More specifically, the unit of analysis was an individual rumor case. The data were coded manually by (1) reading through the data, (2) phasing excerpts to form codes, (3) grouping codes into categories/themes, and (4) interpreting the results. There were no pre-determined lists of codes. All codes arise directly from the data. The researchers developed a list of codes (i.e., what is being said) by reading and re-reading the data (Table 1). This step was iterative and continued until no new codes appear. These codes were then grouped into the following three categories: types of rumors, rumor control agencies, and ways of cooperation (Table 2).

To ensure the trustworthiness of coding, multiple coders are necessary [33]. A team of three coders was used. After coding the items independently, regular meetings were held between them to reach a consensus. The researchers compared and discussed until the codes had at least 70% similarity.

## 3. Results

### 3.1. Types of Rumors

According to the involved subjects, rumors related to public health crises can be divided as viral research, pandemic prevention and control, confirmed cases, overseas pandemic, social welfare, and government action. Diverse types of rumors exhibited distinctive characteristics, thereby requiring different rumor-bunking methods to improve efficiency.

A government-led rumor-debunking model should be adopted against rumors of “confirmed cases” and “government action”. Since these often involve confirmed or suspected cases and the government’s mishandling of the pandemic, these rumors can have a negative impact on social stability, as well as on public perception of the country or the government. Therefore, it is fundamental for the state and the government to issue an authoritative response to these types of rumors.

Alternatively, science-related rumors on “viral research” can be addressed using a scientific community-led approach. The reason is that most of these rumors involve professional knowledge on how the virus is generated, transmitted, and prevented. Therefore, the refutation of these rumors requires the joint effort of a scientific community that is deeply engaged in the professional field, where experts and scholars with professional backgrounds are involved in sharing knowledge and dispelling rumors.

When resolving rumors relevant to “pandemic prevention and control” and “social welfare”, either a rumor-debunking platform-led approach or an approach that involves multiple agents can be adopted. While these types of rumors are closely associated with the public’s daily travel, work, and life, they will not necessarily lead to large-scale social lockdowns or significant impacts on social order. Therefore, the government can entrust this part of the job to rumor-debunking platforms, which can carry out investigations, integrate resources, and combine multiple agents to dispel the rumor using their technology and resource advantages, in order to alleviate the government’s burden and increase the rumor-debunking efficiency.

When dealing with rumors about the progress of overseas pandemic and anecdotes, the government can delegate power to the media and let the media take responsibility of “screeners”. This type of rumor often originates from the curiosity of netizens, who speculate and fabricate rumors out of nowhere. When filtering this kind of rumor, the media can also guide the public and cultivate their rational thinking or promote a more realistic attitude.

### 3.2. Rumor-Debunking Agents

#### 3.2.1. Government Departments

The Chinese collaborative rumor-debunking online platform (www.piyao.org.cn (accessed on 14 March 2021)) divides government-released rumor-debunking information into “ministerial announcements” and “local government responses”; that is, categorizing government departments into central ministries and local governments according to administrative levels. The Chinese government has great information superiority and takes responsibility for public health in China. Most health-related rumors, therefore, have been debunked by the government.

#### 3.2.2. News Media

News media not only serves as a bridge for information transmission between the government and social groups but also acts as a loudspeaker for the government and an acoustic horn for public opinion. Among the selected cases, news media released rumor-debunking information related to official media (such as People’s Daily and CCTV News) and unofficial media (such as Observer.com and The Paper).

#### 3.2.3. Rumor-Debunking Platforms

To gather forces from all sectors of society and successfully dispel rumors, various departments, media, and social organizations have actively established online rumor-debunking platforms. The existing platforms can be divided into three categories. The first category comprises online community rumor-debunking platforms held by online communities, such as the Tengxun Fact Check platform and the Weibo Rumor-debunking platform. The second category comprises regional collaborative rumor-debunking platforms, which are jointly held by local government departments, media, and social organizations, such as the Shanghai Online Rumor-debunking platform and the Beijing Online Rumor-debunking platform. The last category comprises professional rumor-debunking platforms, which are jointly held by social organizations and experts, such as the Kepuchina scientific rumor-debunking platform hosted by the China Association for Science and Technology and DXY.com.

#### 3.2.4. Experts and Scholars

Equipped with innovative knowledge of science and technology, experts and scholars can prevent professional knowledge from being adapted and alienated during dissemination, making them one of the most reliable sources of rumor-debunking information. Experts and scholars can dispel rumors either actively or passively. During passive rumor refutation, experts and scholars first express their opinions in media interviews, which are then published. In contrast, during active rumor refutation, “star experts” who already have a fanbase in the online community, actively respond to rumors by publishing popular science articles on their certified Weibo, WeChat, and other accounts.

#### 3.2.5. Involved Parties

The parties involved in the rumor experience the entire incident and understand the details. Since they are more familiar with the truth, they are an important source of rumor refutation. However, the social influence of these parties is generally small. Therefore, their response to the rumor should be reposted by government departments or news media to reach wider audiences.

### 3.3. Collaboration between Agents

#### 3.3.1. Communication and Verification

Communication and verification refer to the process in which the media acquires rumor-debunking related information through communication, investigation, and verification with government departments, experts, or involved parties. In the process of communication and verification, the government, experts, or involved parties provide authoritative information and the truth, which is then shared with other agents in a timely manner, in order to eliminate information asymmetry and achieve collaborative rumor refutation. “Communication and verification” is one of the primary methods of collaborative rumor refutation that involves multiple agents.

#### 3.3.2. Assisting in Repost

Assisting in repost refers to the process during which the media, rumor-debunking platforms, and netizens repost the rumor-debunking information released by government departments, involved parties, and other agents through various channels, in order to expand the influence of the rumor-debunking information. For specific objects, such as individuals, schools, and enterprises, rumor-debunking information released by the involved party often exhibits little influence and does not reach far, thereby requiring other influential agents to assist in reposting the information. Similarly, “assisting in repost” is one of the main methods of collaborative rumor refutation.

#### 3.3.3. Scientific Collaboration

Scientific health information during the COVID-19 pandemic encompasses blind spots in the public’s knowledge, which makes it more prone to rumors. Scientific communities, such as Kepuchina.com, Yaohuluwa, and DXY.com, have recruited many experts and scholars in the field of scientific health. Therefore, when rumors break out, these communities can publish rumor-debunking information on channels such as WeChat public accounts, Weibo, and websites that have a certain number of followers in order to realize rapid and scientific rumor refutation.

#### 3.3.4. Report and Feedback

The public is the direct recipient of rumors. As their rational thinking, knowledge reserve, and information literacy continuously improve, the public will raise questions about certain rumors and report them to relevant departments, media, and platforms, allowing other agents to note the rumor, intervene, and dispel it.

#### 3.3.5. Resource Integration

During the COVID-19 pandemic, a rumor in place A has often become another rumor in place B, simply by changing the name of the place, but the rumor-debunking information released by various departments and media in place A has not been applicable in place B. To resolve this issue, resource integration breaks the information barrier between different regions, departments, and platforms through information aggregation, in order to promote the optimal allocation of rumor-debunking resources and realize comprehensive rumor refutation.

#### 3.3.6. Invited Collaboration

During a rumor-debunking operation, differences in the roles of the government, the media, experts, social organizations, and netizens determine their different abilities to act. Therefore, regional joint rumor-debunking platforms must constantly invite local government departments, enterprises and institutions, news media, and experts or scholars to join. By taking advantage of the government’s public power advantage, the media’s information dissemination advantage, and experts’ professional advantage, collaborations can categorize the different steps of a rumor-debunking operation and assign them to the most suitable agents, thereby preventing conflicts of actions; on this basis, the efficiency of rumor-debunking can be improved.

## 4. Discussion

### 4.1. Social Collaboration Models

An in-depth analysis of the agents involved in rumor refutation and the collaborative rumor process revealed that there was always a certain agent that led the rumor-debunking operation. This agent was usually in charge of collecting and verifying rumors and eventually releasing the official rumor-debunking information, at which point it would work closely with other agents through various collaborations. This study has summarized five socialized collaborative rumor-debunking models and their main characteristics during the COVID-19 pandemic. On this basis, corresponding measures for these five rumor-debunking models can be implemented to ensure their effective operation, thereby allowing them to address diverse types of rumors in public health crises.

#### 4.1.1. Government-Led Model

**Figure 1:**
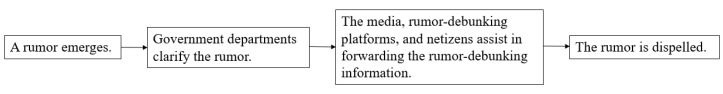
The “government-led” rumor-debunking model.

In the government-led model, government departments play a leading role, while other agents, such as the media and rumor-debunking platforms, play the role of assisting and forwarding information. This model features authenticity (Figure 1). When a rumor emerges and draws public attention, the corresponding clarification issued by related government departments has limited scope of dissemination, due to the traditional communication channels it utilizes. Therefore, the assistance of media, rumor-debunking platforms, and netizens in reposting the rumor-debunking information can broaden the scope of dissemination, thereby facilitating the refutation of the rumor.

The next section covers the following rumor-debunking example: “COVID-19 pandemic outbreaks in Italy. The Chinese embassy in Italy has chartered a flight to evacuate overseas Chinese citizens”.

In March 2020, because of the rapid transmission of COVID-19 in Italy, many overseas Chinese citizens were eager to return to China, which led to the following rumor: “The Chinese embassy in Italy is preparing to charter flights to evacuate overseas Chinese citizens”(stage 1: a rumor emerged). On March 6, when asked by reporters, the spokesperson of the Chinese embassy in Italy clarified that the embassy never considered chartering flights to evacuate overseas citizens. Subsequently, an official rumor-debunking announcement was published on the website of the Chinese embassy in Italy (stage 2: government departments clarified the rumors). However, this information was not effectively disseminated because of the limited influence of the embassy’s website. Fortunately, other media channels, including Chinanews.com, Huanqiu.com, and Guancha.cn, reposted the embassy’s rumor-debunking information on their websites, WeChat public accounts, official Weibo, and other channels, and it then became trending news on Weibo (stage 3: the media, rumor-debunking platforms, and netizens assisted in forwarding the rumor-debunking information). The rumors of evacuating overseas Chinese citizens, therefore, was dispelled.

The implementation of the government-led model requires a comprehensive information disclosure system, as well as broad information dissemination channels. Since Chinese people have a prominent level of trust in the government, authoritative, accurate, and timely disclosure of government information is an effective way to stop rumors from spreading [31]. Therefore, the government should normalize and institutionalize information disclosure, report confirmed cases and treatment conditions in a timely manner and explain relevant prevention or control policies and countermeasures. Furthermore, in addition to utilizing official channels, such as government press conferences, official websites, Weibo, and WeChat official accounts, the government can actively collaborate with credible news media and online platforms to exploit their information dissemination advantage by asking them to forward the authoritative rumor-debunking information. At the same time, the government should continuously monitor the progress of each rumor, promptly release authoritative information-debunking information, and utilize mass information against the explosive transmission of rumors. In addition, the government may take responsibility for forming a multi-organization mechanism that integrates social organizations, media, health professionals, and the public.

#### 4.1.2. Media-Led Model

**Figure 2:**
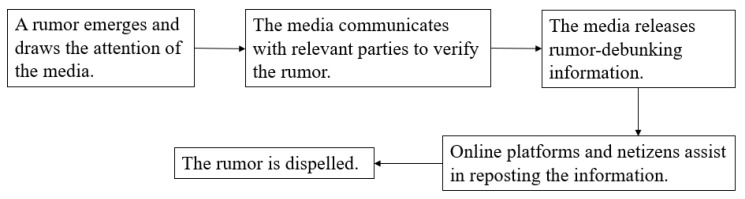
The “media-led” rumor-debunking model.

In the media-led model (Figure 2), the media plays a dominating role, whereas government departments and parties act as sources of information, and platforms and netizens participate by reposting information. When a rumor emerges and gains public attention, the media can utilize its resources to quickly contact relevant departments and parties and verify the rumor, before releasing rumor-debunking information. This model has the advantage of being instantaneous.

The next section covers the following rumor-debunking example: “Smokers are far less likely to be infected with COVID-19 than non-smokers”.

On 12 February 2020, a WeChat public account named “Yao Talk Lessons” posted an article titled “Smokers are far less likely to be infected with COVID-19 than non-smokers? Nanshan Zhong publishes his first paper”, which not only suggested that “The COVID-19 infection rate among smokers is substantially lower than that among non-smokers” but also claimed that smoking could exorcise evil and cure diseases. By noon on 13 February, the article reached “100,000+” reads and was extensively reprinted (stage 1: a rumor emerged). On 13 February, a reporter from Beijing Youth Daily noticed the article and questioned the authenticity of the information it presented. By interviewing Jianshu Zhang, president of the Beijing Tobacco Control association, and contacting Nanshan Zhong’s team, the reporter then confirmed that the paper published by Nanshan Zhong’s team did not include the conclusion that “The COVID-19 infection rate among smokers is substantially lower than that among non-smokers.” (stage 2: the media communicated with relevant parties to verify the rumor). Subsequently, the Beijing Youth Daily published the corresponding rumor-debunking news (stage 3: the media released rumor-debunking information). Meanwhile, a few other media also confirmed that the claim that “Smokers are far less likely to be infected with COVID-19 than non-smokers” was a rumor, by consulting experts and scholars in relevant institutions (here, we can observe that more than one media worker was involved in stage 2). In the end, associated rumor-debunking articles released by multiple media, such as Beijing Youth Daily, were reposted by the Chinese collaborative rumor-debunking online platform (www.piyao.org.cn (accessed on 14 March 2021)), the Weibo rumor-debunking platform, and many netizens (stage 4: online platforms and netizens assisted in reporting the information), thereby successfully dispelling the rumor (stage 5).

In the media-led rumor-debunking model, the media catches wind of rumors reported by the public, then communicates and verifies them with government departments, experts, and social organizations, and finally transmits the rumor-debunking information to the audience through its communication channels (at which point it acts as an information hub). The public also tends to use social media to seek credible health information from the government or social organizations [34]. Therefore, the media should fully utilize its resource advantages, monitor changes in social public opinions in real time, and capture rumors promptly. For example, the media may contribute to various forms, such as pictures and texts, short videos, and special columns. In addition, it should strengthen communication with the public to engage them to also monitor rumors. Another way for the media to facilitate rumor refutation is to construct an information sharing and data exchange platform to improve the information exchange between the media and other agents and establish a three-dimensional multi-source information chain.

#### 4.1.3. Scientific Community-Led Model

**Figure 3:**
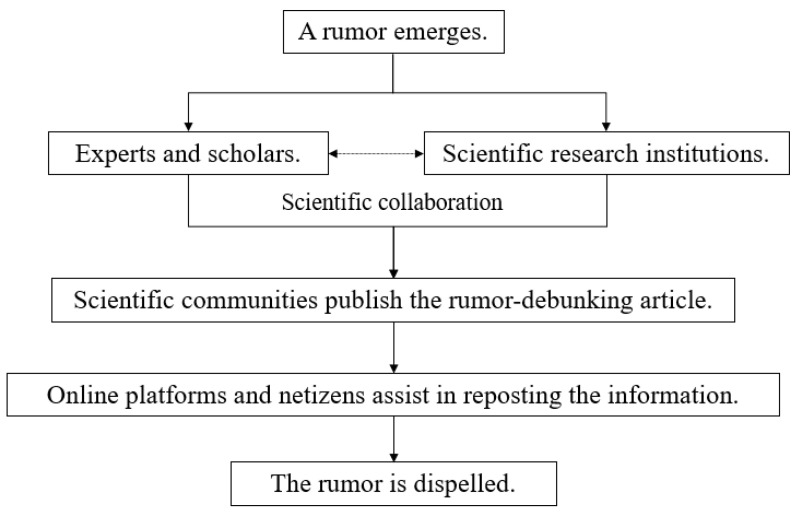
The “scientific community-led” rumor-debunking model.

The scientific community-led model is guided by the scientific community, which is composed of scientific research institutions, experts, and scholars in relevant fields. In this model (Figure 3), the power of experts and scholars is gathered by exchanging knowledge within the community and establishing a professional rumor-debunking alliance. The model acquires rumor-debunking information through means such as knowledge exchange, joint publication, and mutual reviews, which are then followed by releasing rumor-debunking articles on its accounts. Therefore, it features the advantage of being scientifically viable.

The next section covers the following rumor-debunking example: “Antibiotics can prevent COVID-19 infection.”

Kepuchina.com is a popular science platform hosted by the China Association for Science and Technology, which has recruited experts in food, health, medical, and other fields and has set up dedicated rumor-debunking sections. During the early days of the COVID-19 outbreak, rumors that antibiotics such as “azithromycin”, “moxifloxacin”, and “cephalosporin” could prevent and treat COVID-19 were circulating on the Internet (stage 1: a rumor emerged). Therefore, Kepuchina.com consulted Guiyang Liu, the chief pharmacist of the Fourth Medical Center of the People’s Liberation Army General Hospital, and Jin Liu, the chief pharmacist of the Department of Pharmacy of the China-Japan Friendship Hospital (stage 2: the collaboration happened between scientific communities). Through mutual verification of the two experts’ opinions, the platform released rumor-debunking information (stage 3: scientific community published the rumor-debunking article). This was subsequently reposted and disseminated by multiple media channels and netizens and successfully dispelled (stage 4: online platforms and netizens assisted in reposting the information and stage 5: the rumor was dispelled).

The field of science is not only closely related to public health and daily life, but it is also the “most impacted area” by rumors during public health crises. In the scientific community-led rumor-debunking model, experts and scholars often disseminate scientific knowledge to the public unidirectionally, while the latter passively absorbs the rumor-debunking information due to insufficient knowledge. It is necessary to use appropriate measures to educate the public on how to prevent health-related rumors in their daily life. The education courses should be easy to understand and avoid using professional terminology and preaching. Research shows that engaging the public in controlling rumors has significant benefits, and they are happy to spread credible information to debunk rumors [35,36]. Therefore, it is important to improve public information literacy and set up scientific communication channels. As a result, when the public discovers a scientific rumor, they can learn relevant scientific knowledge through search tools and database resources, thereby forming a bidirectional interactive science communication environment.

#### 4.1.4. Rumor-Debunking Platform-Led Model

**Figure 4:**
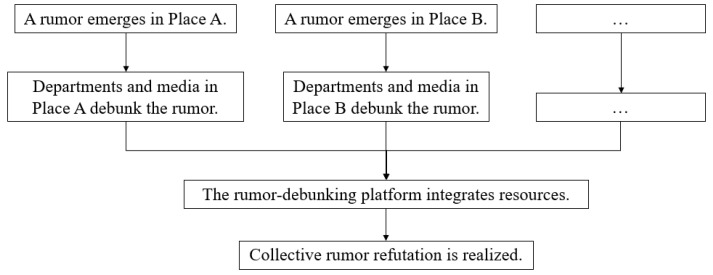
The “rumor-debunking platform-led” rumor-debunking model.

The rumor-debunking platform-led model, which is primarily guided by rumor-debunking platforms, integrates the rumor-debunking information released by different departments, media channels, and experts in various regions to realize collective rumor refutation (Figure 4). Therefore, it maintains integrity and takes a systematic approach. In the early days of the COVID-19 outbreak, rumors such as “Three people are not allowed to travel together” and “Air Force will send planes to spray disinfectant powder” emerged. After the pandemic situation improved, rumors on the timeline for removing masks and resuming schools, as well as the nationwide suspension of passports, began to spread. In this context, when there is an overwhelming amount of true and false news, official rumor-debunking information is sometimes lost. Therefore, a rumor-debunking platform-led system that collects clarifications on local rumors released by departments and media platforms is required to eliminate the regional barrier of disseminating rumor-debunking information.

The next section covers the following rumor-debunking example: “Timetable for removing masks in 31 provinces and cities across China has been determined”.

With improvements in the pandemic situation, rumors about the timeline for removing masks emerged in various places. At the end of April 2020, a “timetable for removing masks in 31 provinces and cities across China” was widely circulated on the Internet, attracting national attention. This table was a mixture of rumors from various places in China (stage 1: a similar rumor emerged in many places). Between 24 and 29 April, relevant local government departments in Chengdu, Chongqing, Shanghai, Hebei, and Shenzhen successively pointed out that the “timetable for removing masks across China” was fake news (stage 2: the rumor was debunked in various places). In addition, experts, and scholars, such as Yi Shi, a researcher at the Institute of Microbiology of the Chinese Academy of Sciences, Liubo Zhang, a researcher at the Chinese Center for Disease Control and Prevention, and academic named Nanshan Zhong, all pointed out that it was too early to remove masks. As a result, the Chinese collaborative rumor-debunking online platform (www.piyao.org.cn (accessed on 14 March 2021)) integrated rumor-debunking information from various departments, experts, and scholars and published articles for collective rumor refutation (the final two stages in Figure 4).

During the COVID-19 pandemic, diverse types of rumors have emerged and have filled up rumor-debunking platforms across different regions. Therefore, how to integrate recurring rumors at various places and corresponding rumor-debunking information is critical for dispelling rumors. At present, the platforms have their own rumor-refuting channels and related mini-programs, but the databases of each platform are still relatively fragmented, and a collaborative mechanism has not yet been formed. The rumor-debunking platform-led rumor-debunking model also suffers from fragmented information from different areas and platforms, making it necessary to establish a real-time, simple, open-source, community-centered rumor-debunking database [37]. This database can then classify recurring rumors of the same type and identify their origin. Meanwhile, it is necessary to establish a comprehensive rumor-refuting mechanism that integrates new platforms and traditional platforms. For example, a national-level rumor management big data platform that integrates websites, newspapers, radio, TV, and social organizations can be built in the future.

#### 4.1.5. Multi-Agent Collaborative Model

**Figure 5:**
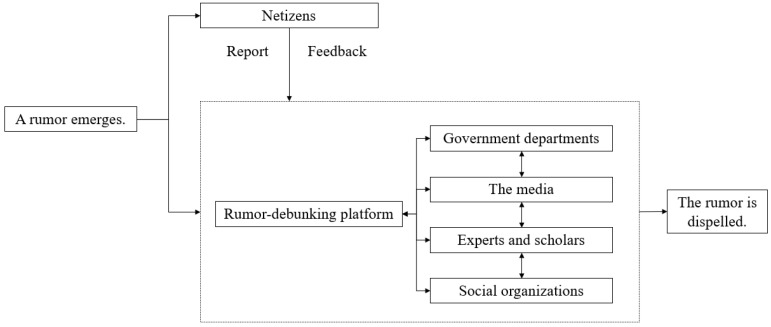
The “multi-agent collaborative” rumor-debunking model.

In the multi-agent collaborative model, several agents, including government departments, the media, rumor-debunking platforms, experts, and the public, jointly participate in rumor-debunking operations (Figure 5). With rumor-debunking platforms as the carrier, the model combines powers of the official and the private, as well as the central and the local sectors, thereby being authentic, instantaneous, scientific, and systematic, and upholding integrity. The emergence of rumor-debunking platforms has enabled the collaboration of multiple agents, promoting the transition of the rumor-debunking model from the traditional path of “rumor emerges–government and media dispel the rumor” to “rumor emerges–users report the rumor–the rumor is dispelled jointly”.

The next section covers the following rumor-debunking example: “If you leave Wenzhou during the Labor Day holiday, you must undergo PCR testing at your own cost upon your return”.

At the end of April 2020, an article claiming that “According to Notice No. 88 of the Wenzhou COVID-19 Pandemic Prevention and Control Leading Group, during the Labor Day holiday, residents are advised against leaving the city. Those who leave the city are required to undergo PCR and serum tests upon returning at their own expenses” was circulating in Wenzhou citizens’ WeChat groups and WeChat moments (i.e., a rumor emerged). Multiple netizens left messages on the “Q&A” section of the Wenzhou rumor-debunking platform, asking whether the notice was true (i.e., netizens reported the rumor). By reviewing notices of Wenzhou City and Zhejiang Province on the pandemic prevention and control measures during the Labor Day holiday, the Wenzhou pandemic Prevention and Control Office and the Zhejiang pandemic Prevention and Control Office confirmed that it was fake news. Subsequently, the Wenzhou rumor-debunking platform refuted the rumor through multiple communication channels, including websites, Weibo, and WeChat official accounts (i.e., multiple agents collaborated to dispel the rumor).

This kind of rumor-debunking in which netizens first ask questions about the rumor on the rumor-debunking platform and gain the attention of relevant departments, forcing them to respond, clearly demonstrates the closed-loop development of the multi-agent collaborative rumor-debunking model. This model significantly escalates the efficiency of rumor refutation by improving the interaction between agents.

The control of health-related rumors requires cooperation between the government, the media, rumor-debunking platforms, experts, and the public [34]. Different agents of socialization have different interest demands. Therefore, effectively understanding and coordinating the interest relationship between various agents, as well as maximizing complementarity and seeking advantages while avoiding disadvantages, is the basis for operating a multi-agent collaborative rumor-debunking model.

First, it is necessary to establish a community of public interests, promote a consensus in terms of social value orientation, and strengthen the sense of belonging and identity of various agents in the collaborative rumor-debunking operation, to improve the interaction and cooperation of different rumor-debunking agents. Second, as the organizer and guide of the collaborative rumor-debunking operation, the government can formulate interest coordination policies, establish an interest communication mechanism, and provide channels for interest communication and negotiation, to ensure the effective operation of the socialized collaborative rumor-debunking mechanism. Third, an incentive system should be implemented. Media, social organizations, experts, and the public that actively dispel rumors should be provided with spiritual or material rewards, in order to fully promote the initiative among all agents. Last, media literacy plays a significant role in debunking health-related rumors; it prevents the appearance and spread of rumors. Media literacy is the ability to access, analyze, evaluate, and create information across all forms of media [38]. It usually involves increasing skills and building knowledge [39]. To prevent health-related rumors, governments or experts should actively educate the public through all kinds of media literacy-related initiatives (for example, the Metro Toronto Movement for Literacy (MTML) in Canada and the Digital Literacy Initiatives in the United States). These initiatives can reach wider audiences with the help of the media. Meanwhile, the public should be actively involved in these initiatives to enhance their ability to distinguish information.

### 4.2. Contribution of the Study

This study has its significance. Past research has studied how to debunk rumors. Some scholars focus on developing and examining the specific rumor-debunking methods. For example, Rubin noticed that rumors on social media can be debunked with common sense judgements, further investigations by professionals, and rumor detection systems [40]. Liu et al. introduced the first real-time rumor debunking algorithm for social media [41]. Wang et al. divided rumor debunking methods into six categories (i.e., denial, further fact-checking, refutation, person response, organization response, and combination methods), of which the refutation method has the best debunking effect [42]. Other researchers tried to find out influencing factors related to rumors. For instance, Merlino and Tabasso found that whether individuals check messages/information is related to their view of the world. In other words, people are less likely to check the message in line with their bias. Consequently, they proposed that the success of debunking rumors requires incentivizing individuals to verify the information [43]. Song et al. examined how rumor types, content attributes, and source characteristics affect the likelihood of sharing rumors [44]. These existing studies represent significant contributions to guiding rumor-debunking in practice. However, they have paid much attention to a single group, neglecting the role of multiple agents in debunking rumors. This study furthers the existing research by emphasizing the collaboration of various agents. Debunking rumors, especially health-related rumors, requires the cooperation of different groups rather than solely relying on one specific group. After all, no one can whistle a symphony.

By theoretically expanding the research perspective of involving multiple agents in rumor debunking, this study can add related research on collaborative rumor refutation. In addition, the study verifies the value of socialized collaborative rumor debunking, advocates and encourages the participation of multiple agents of socialization, and provides guidance for establishing a collaborative rumor-debunking model, thereby promoting efficient rumor-debunking methods and improving the healthcare of society.

### 4.3. Limitations of the Study

This study has some limitations. First, it adopted rumor-debunking cases during the COVID-19 pandemic as its research object. However, for public health crises that have a small impact, occur in local areas, or last for a short duration, more work is required to refine corresponding rumor-debunking models. Second, the practical suggestions we proposed have not yet been examined in practice. More research is needed to examine and develop these models. Finally, the models in this paper were proposed based on health-related rumors in China. Considering the influence of Chinese history and culture, these models might not be directly applied to other countries. More efforts are required in follow-up studies to enrich the research material and deepen the research process, to further optimize and improve related research.

## 5. Conclusions

During the COVID-19 pandemic, a considerable number of health-related rumors have been found, which have prevented people from complying with protective behavior (e.g., wearing masks and keeping social distance), undermined trust in healthcare providers, and threatened public health [37]. The emergence of online media has worsened this situation, since it has accelerated the speed of information dissemination and expanded its scope, thereby increasing the difficulty of rumor refutation. The most effective and direct way against rumors is to dispel the rumor in a timely manner and curb its spread, thereby returning the truth to the public. In the era of social media, a single agent cannot effectively handle all types of Internet rumors during public health crises. The rise of social media has also facilitated the joint participation of multiple social forces in rumor-debunking operations, and multiple agents must be engaged in the future to dispel rumors.

This study investigated the main compositions and coordination of socialized rumor-debunking models. The study found that agents of socialization play a crucial role in rumor refutation during the COVID-19 pandemic, demonstrating a higher participation in rumors related to viral research, confirmed cases, and social welfare. Six collaborative relationships among different agents were identified during the rumor-debunking process, which are as follows: communication and verification, assisting in reposting, scientific collaboration, report and feedback, resource integration, and invited collaboration. In addition, socialized rumor-debunking models could be divided into the following five categories: the government-led model, the media-led model, the scientific community-led model, the rumor-debunking platform-led model, and the multi-agent collaborative model. At the same time, since rumors in public health crises often involve different objects, rumor refutation requires various information sources; therefore, different rumor-debunking models apply. For example, experts are the source of authoritative information on rumors about how the virus spreads and how to protect and treat it. Therefore, they are more able to convince the public through the scientific community-led model, i.e., the cooperation of scientific community and experts. In response to rumors that involve local epidemic prevention and control measures, confirmed cases, social life, etc., local government departments are required to provide authoritative information in a timely and proactive manner, and then media, platforms, and netizens can assist in forwarding the information. These rumor-refuting models require a series of measures to ensure their effective operation, which can promote deep collaboration between different agents, overcome the shortcomings of the existing rumor-debunking models, and exert the effect of collaborative rumor refutation, thereby jointly creating a clear cyberspace.

## Figures and Tables

**Table 1:** Examples of coding.

Data	Codes
People’s Daily verified the authenticity of the news that “Huoshenshan Hospital was blown away overnight” with the involved hospital.	Pandemic prevention and control Involved party News media Communication and verification
Life Times consulted Professor Zhanqiu Yang from the School of Medicine, Wuhan University, about the authenticity of the news that “COVID-19 spreads exclusively among Chinese or East Asian people.”.	Pandemic prevention and control News media Experts and scholars Viral research
Hubei Daily, Huanqiu.com, and other media reposted a statement from the Suizhou Internet Police on the refutation of the rumor that “An entire family in Suizhou, Hubei, has developed world-weariness after catching COVID-19 and therefore started throwing money from their home.”.	Assisting in repost News media Government department Social welfare
Wei Li, Chief Pharmacist of the Zhengzhou Maternal and Child Health Hospital, authored an article, which was reviewed by Baoxin Wang, Deputy Chief Pharmacist of the Peking University First Hospital, to jointly refute the rumor that “Low temperature can kill the COVID-19 virus.”.	Scientific collaboration Experts and scholars Viral research
Qianlong.com, Haidian news, and other media reposted a statement from the Beijing Shangdi Innovation Building on the refutation of the rumor that “The Beijing Shangdi Innovation Building is locked down because multiple employees have developed fever symptoms and must be isolated.”.	Assisting in repost Involved party News media Confirmed cases
Netizens reported that some people are spreading news that “An old man has committed suicide after being punished by the police to write down ‘I must wear a mask when going outdoors’ a hundred times”, which was confirmed to be a rumor by the Zhonglou Police Station.	Report and feedback Government department Social welfare
Netizens reported the spreading of associated rumors, including “If you leave Wenzhou during the Labor Day holiday, you must undergo a PCR test at your own cost upon your return” on the Wenzhou rumor-debunking platform, which was jointly refuted by Wenzhou Pandemic Prevention and Control Office, the Zhejiang Provincial Pandemic Prevention and Control Office, and other departments.	Invited collaboration Government department Rumor-debunking platform Pandemic prevention and control

**Table 2:** Three code categories.

Categories	Codes
Types of rumors	Viral research Pandemic prevention and control Confirmed cases Overseas pandemic Social welfare Government action
Rumor control agencies	Government department News media Rumor-debunking platform Experts and scholars Involved party
Ways of cooperation	Communication and verification Assisting in repost Scientific collaboration Report and feedback Resource integration Invited collaboration

## Data Availability

The data presented in this study are available upon request from the corresponding author.
